# Corrections officers’ knowledge and perspectives of maternal and child health policies and programs for pregnant women in prison

**DOI:** 10.1186/s40352-019-0102-0

**Published:** 2020-01-04

**Authors:** Virginia Pendleton, Jennifer B. Saunders, Rebecca Shlafer

**Affiliations:** 1 Division of Epidemiology, School of Public Health, University of Minnesota, 420 Delaware Street SE, Minneapolis, MN 55455 USA; 2 Division of Health Policy and Management, School of Public Health, University of Minnesota, 420 Delaware Street SE, Minneapolis, MN 55455 USA; 3 Division of General Pediatrics and Adolescent Health, University of Minnesota, 717 Delaware Street SE, Minneapolis, MN 55414 USA

**Keywords:** Corrections officer, Prison, Pregnancy, Maternal and child health, Doula, Mixed methods

## Abstract

**Background:**

In response to the dramatic increase in the number of women incarcerated in the United States—and a growing awareness that a small proportion of women enter prison pregnant and have unique health needs—some prisons have implemented policies and programs to support pregnant women (defined here as maternal and child health [MCH] policies and programs). Corrections officers (COs) are key stakeholders in the successful implementation of prison policies and programs. Yet, little empirical research has examined prison COs’ knowledge and perspectives of MCH policies and programs, particularly the impact such policies and programs have on COs’ primary job responsibility of maintaining safety and security. The objective of this mixed-methods study was to understand COs’ knowledge and perspectives of MCH policies and programs in one state prison, with a specific emphasis on the prison’s pregnancy and birth support (doula) program.

**Results:**

Thirty-eight COs at a single large, Midwestern women’s prison completed an online survey, and eight of these COs participated in an individual, in-person qualitative interview. Results indicated that COs’ perspectives on MCH policies and programs were generally positive. Most COs strongly approved of the prison’s doula program and the practice of not restraining pregnant women. COs reported that MCH policies and programs did not interfere, and in some cases helped, with their primary job task of maintaining safety and security.

**Conclusions:**

Findings support expansion of MCH programs and policies in prisons, while underscoring the need to offer more CO training and to gather more CO input during program development and implementation. MCH services that provide support to pregnant women that are outside the scope of COs’ roles may help reduce CO job demands, improve facility safety, and promote maternal and child health.

## Background

The United States (US) has the largest population of incarcerated women in the world, with approximately 112,000 women in federal or state prisons and another 110,000 in jails (Carson 2018; Walmsley 2017). Women in prison have health care needs unique from incarcerated men, including gynecological and obstetric services (Tapia and Vaughn 2010). National data on pregnancy in prisons are not routinely collected, but a recent study estimates that 3.8% of newly admitted women in US prisons are pregnant (Sufrin et al. 2019). Although some of these women are released before giving birth, each year an estimated 1400 women in the US give birth while incarcerated (Sufrin et al. 2019). As the female prison population has risen, there has been a growing call for gender-responsive policies and programs in carceral settings, including the development and implementation of programs that meet the unique needs of pregnant women in prison (Covington and Bloom 2007; Goshin et al. 2017; Sufrin 2017).

Policies and programs that aim to support the health of pregnant women have been implemented in some prisons across the country (Baldwin et al. 2018; Ferszt et al. 2013). Using a public health framework, this paper defines services that seek to support the mental, physical, and emotional health of pregnant women in prison and their children as “maternal and child health (MCH)” programs and policies. Such programs include supplemental nutrition, modifications to pregnant women’s daily living and work routines, anti-shackling policies, adoption and abortion services, doula programs, parenting classes, breastfeeding support, and prison nurseries (Baldwin et al. 2018; Saar 2010).

A handful of states have programs specifically for pregnant and laboring incarcerated women (Froggé 2019). For example, in some Minnesota jails and prisons, the Minnesota Prison Doula Project (MnPDP) provides weekly parenting classes and one-on-one doula support to incarcerated women [for more information on the MnPDP see (Shlafer et al. 2014)]. In many prison systems, including Minnesota, women in labor are not allowed to have family members or friends attend the birth (Fritz and Whiteacre 2016; Mason 2013). As a person “trained and experienced in childbirth who provides continuous physical, emotional, and informational support to the mother before, during and just after the birth,” doulas provide in-person support that pregnant women in prison cannot receive from family members or friends during labor and delivery (Doula Organization of North America 2017). Unlike doctors or midwives, doulas do not provide medical support or have clinical responsibilities; instead they offer physical comfort, give reassurance through emotional support, and use lay language to describe what is happening throughout the labor. Through the MnPDP, a doula typically meets with a pregnant woman at the prison twice before birth, attends her labor and delivery, is present on the day the woman and her infant are separated when the woman returns to prison, and meets twice after the birth [Shlafer et al. 2014].

Many states have also modified existing policies to address the unique needs of pregnant women. One specific policy that has garnered much attention over the past decade is the policy against restraining or “shackling” pregnant women with handcuffs or other devices that limit movement (CBS News 2019; Ferszt et al. 2018). Medical contraindications to the use of restraints include interfering with balance and increasing the risk of falls, causing delays during medical emergencies, limiting mobility which can make labor more difficult, and impeding mother and infant bonding (Ferszt et al. 2018; American College of Obstetricians and Gynecologists 2012; Shackling of incarcerated pregnant women: AWHONN’s position 2012). The Federal Bureau of Prisons has prohibited shackling of pregnant women in all federal facilities since 2008 (Ferszt et al. 2018). As of 2018, 22 states and the District of Columbia had some form of state anti-shackling legislation (Ferszt et al. 2018; King 2018).

There are numerous stakeholders in successful implementation of MCH policies and programs in carceral settings, including prison administrators, health services staff, corrections officers (COs), community-based organizations, and incarcerated pregnant women. Previous research has examined the knowledge and perceptions that some of these key stakeholders have of MCH policies and programs for pregnant women in prison (Campbell and Carlson 2012; Ferszt and Clarke 2012; Fritz and Whiteacre 2016; Schroeder and Bell 2005a, b; Shlafer et al. 2014; Williams and Schulte-day 2006; Wismont 2000). However, the majority of this research has been with health care providers, particularly nurses (Ferszt et al. 2013; Goshin et al. 2019; Zust et al. 2013), and other key stakeholders have been considered less often.

COs hold an integral position within the carceral system and are key personnel in the successful implementation of new policies and programs (Ferszt and Erickson-Owens 2008; Lambert et al. 2018). A primary component of CO’s job responsibilities is to ensure the “safety, accountability, welfare and security of the general public, facility, on-site personnel, and offenders” (Minnesota Department of Corrections 2019). In addition to their typical duties and interactions with pregnant women in the prison, COs are also present at a woman’s delivery at the hospital. At the hospital, COs’ responsibilities are to maintain the safety and security of the general public, on-site personnel (e.g. medical staff), and the pregnant women under their custody (Kelsey et al. 2017; Wismont 2000).

Supporting women in labor is not typically part of a COs’ job, and physical touch or emotionally engaging with incarcerated people in their care is often prohibited through prison policies (Halsey and Deegan 2017; Wismont 2000). Within the correctional setting, boundary violations are violations of the rules and regulations that are put in place to maintain professional distance between COs and people in prison (Marquart et al. 2001). Despite their substantial role in supervising pregnant women in prison and during labor and delivery at the hospital, COs’ knowledge and perceptions of MCH programs, and how these programs may impact COs’ ability to maintain professional boundaries and perform their essential job responsibilities, are largely unknown.

As the largest occupational group in prisons, COs have unique perspectives and influence on the success of any new MCH policy and program (Ferszt and Erickson-owens 2008). As carceral facilities increasingly recognize the unique needs of pregnant and parenting women and incorporate MCH policies and programs into their services, it is important to implement services that are feasible while having the greatest positive impact for women. Ferszt and Erickson-Owens (2008) evaluated the development of an educational group for pregnant women in prison, and found that successful implementation required CO buy-in. MCH policies and programs that increase job demands for COs likely result in increased job stress (Lambert et al. 2018). Further, high levels of job stress correlate with poor health effects for COs and have consequences for overall staffing at the prison, such as increased absenteeism, premature retirement, and high turnover (Armstrong and Griffin 2004; Finn 1998).

The objective of this mixed-methods study is to understand COs’ knowledge and perceptions of programs and policies that support pregnant women in prison, with a specific emphasis on understanding COs’ perceptions of the MnPDP, a unique MCH program at the prison in which the research was conducted.

## Method

### Setting

This study was conducted with COs at one state prison in the Midwest where one of the longest-running prison doula programs in the county, the MnPDP, has been operating since 2010. This facility is the state’s only prison for women, and houses approximately 600 women at all security levels [Minnesota Department of Corrections 2019]. The MnPDP offers pregnancy classes, parenting classes, and doulas (non-medical support) to pregnant women before, during, and after delivery. Since the MnPDP started, more than 100 women at the prison have received doula support through the program. Women are typically transported by two COs to a local community hospital to receive prenatal care during the later stages of pregnancy and to give birth. Women at the prison are not allowed to have family members or friends present during the labor and delivery, and are not allowed contact with visitors throughout their hospital stay, similar to other facilities in the U.S. (Fritz and Whiteacre 2016; Shlafer et al. 2014).

### Procedures and participants

This is a mixed-methods study; an online survey was used to collect quantitative data from COs and in-person interviews were conducted to gain a deeper understanding of COs’ perspectives. A prison administrator directly invited all COs at the prison (*N* = 137) to participate in the study via email with a link to the anonymous, online survey. All survey collection took place over a three-week period in June 2018. Of the 137 COs working at the prison at the time the survey was emailed, 38 (28%) completed the survey.

At the end of the anonymous survey, COs were invited to complete a separate online form to indicate their interest in participating in an in-depth, individual interview about their experience working with pregnant women. Nine COs originally expressed interest in participating in the individual interview, and eight COs (21% of the survey respondents) participated in the interview. All individual interviews were conducted by the principal investigator (RS) in a private space at the prison during COs’ work hours arranged in coordination with the prison administrator. The interviews were audio recorded and ranged from 23 min to 53 min (*M =* 37 min). The interviews took place over a four-week period in the fall of 2018.

Participants did not receive compensation for completing the survey or interview. The Institutional Review Board at the [University of Minnesota] and Human Subjects Review Board at the [Minnesota] Department of Corrections approved this study.

### Measures

The survey consisted of 91 items that asked COs to report on their demographic characteristics, job stress, knowledge and perspectives of the programs and policies available to pregnant women in prison, and training received about working with pregnant women. Job stress-related questions were informed by Armstrong and Griffin’s (2004) definition of job stress as “any disturbance of an individual’s physiological, psychological, or social functioning in response to a condition in the work environment which poses a perceived threat to an individual’s well-being or safety.” The semi-structured individual interviews were intended to complement the survey results, and included the same general topics as the survey. COs discussed their knowledge and perspectives on policies and programs available to pregnant women at the prison and whether policies had changed over time. Because this study was in part a program evaluation of the MnPDP, a substantial portion of the questions asked specifically about the MnPDP including how the program influenced COs’ job responsibilities and what effect COs believed the program has had on their work, pregnant women, and their infants.

### Data analysis

Descriptive statistics were calculated to summarize CO demographics and responses to survey items. Survey responses were analyzed with SPSS v.25. The eight recorded interviews were transcribed and checked for accuracy, and were coded for themes using NVivo Pro 12. An iterative process of coding development followed; all interviews were first independently coded by two researchers (VP and JS). A coding comparison query was conducted for all coding themes in NVivo Pro 12. The Cohen’s Kappa coefficient was used to examine inter-rater reliability to ensure reliability. Coding themes with Kappa coefficient values of ≤0.75 were reviewed and discussed between the two investigators, and coding was adjusted as needed to improve reliability. After primary codes were established, the primary author led the development of themes through qualitative content analysis, which were then refined and agreed upon by all investigators.

To provide perspective into the potential biases introduced in data collection and analysis, the investigators took time to reflect on their own background knowledge and perspectives. All three investigators were white women at the (University of Minnesota) who generally supported MCH programs and policies, including doula programs, being available to women in prison. Engaging in this reflective practice, having two investigators who did not conduct the interviews code the qualitative interviews and compare codes for reliability, and triangulating the results of the qualitative interviews with the quantitative survey results, helped control for potential bias when analyzing results.

## Results

Demographic characteristics of survey and interview participants are summarized in Table 1. The gender, race, ethnicity, and number of years worked as a CO for the participants in this study generally reflected the demographics of the total CO population at the prison (Bosch, G., personal communication, May 20, 2019). Most COs who completed the survey (58%) reported having attended a birth in their role as a CO. The number of births attended over their tenure ranged widely, but most COs had attended five births or less (*Range* = 0–50, *Median* = 3).

**Table 1:** Demographic characteristics of corrections officers who participated in the survey and interviews

	Survey participants (*n* = 38)	Interview participants (*n* = 8)
Characteristic	%	%
Age		
18 to 34 years old	47.3	25.0
35 to 44 years old	21.1	25.0
45 to 54 years old	26.3	37.5
55 years and older	5.3	12.5
Gender		
Male	44.7	37.5
Female	55.3	62.5
Race		
Asian	0	0
American Indian or Alaska Native	0	0
Black or African American	2.6	12.5
Native Hawaiian or Other Pacific Islander	0	0
White	97.4	87.5
Multiracial	0	0
Ethnicity*		
Hispanic or Latino/a	0	0
Non-Hispanic or Latino/a	100	100
Highest level of education completed		
High school or GED	18.4	25.0
Associate’s degree	23.7	25.0
Bachelor’s degree, master’s degree, doctorate or other professional degree (e.g. MD, JD)	57.9	50.0
Parent or caregiver		
Parent or caregiver to one or more children, including step-children or foster children	55.3	50.0
Not a parent or caregiver	44.7	50.0
Years worked as a corrections officer		
< 1 to 3 years	28.9	12.5
4 to 10 years	23.7	25.0
11 to 20 years	31.6	50.0
> 20 years	15.8	12.5

Notes: *One participant did not report their ethnicity. All percents reported are valid percents

Compared to the survey sample, interview participants were more likely to be women, older in age, and had a lower level of post-secondary education. COs who participated in the interviews had worked as COs from 3 to 28 years (*M =* 14 years, *Median* = 14.5 years). COs who participated in the interviews reported attending a wide-ranging number of births (*Range* = 0–40; *Median =* 2).

### Survey participants’ knowledge of policies and programs for pregnant women

A majority of COs who completed the survey identified that MCH policies and programs were available to pregnant women at the prison (see Fig. 1). COs had less awareness of some specific MCH programs; relatively fewer COs were aware of pregnant women’s access to abortion counseling and services (54%) or breastfeeding support (46%). Although 92% of COs reported they were aware of the prison doula program, more than one-third (38%) reported they were “not at all familiar” with the specifics of the program.

**Fig. 1:**
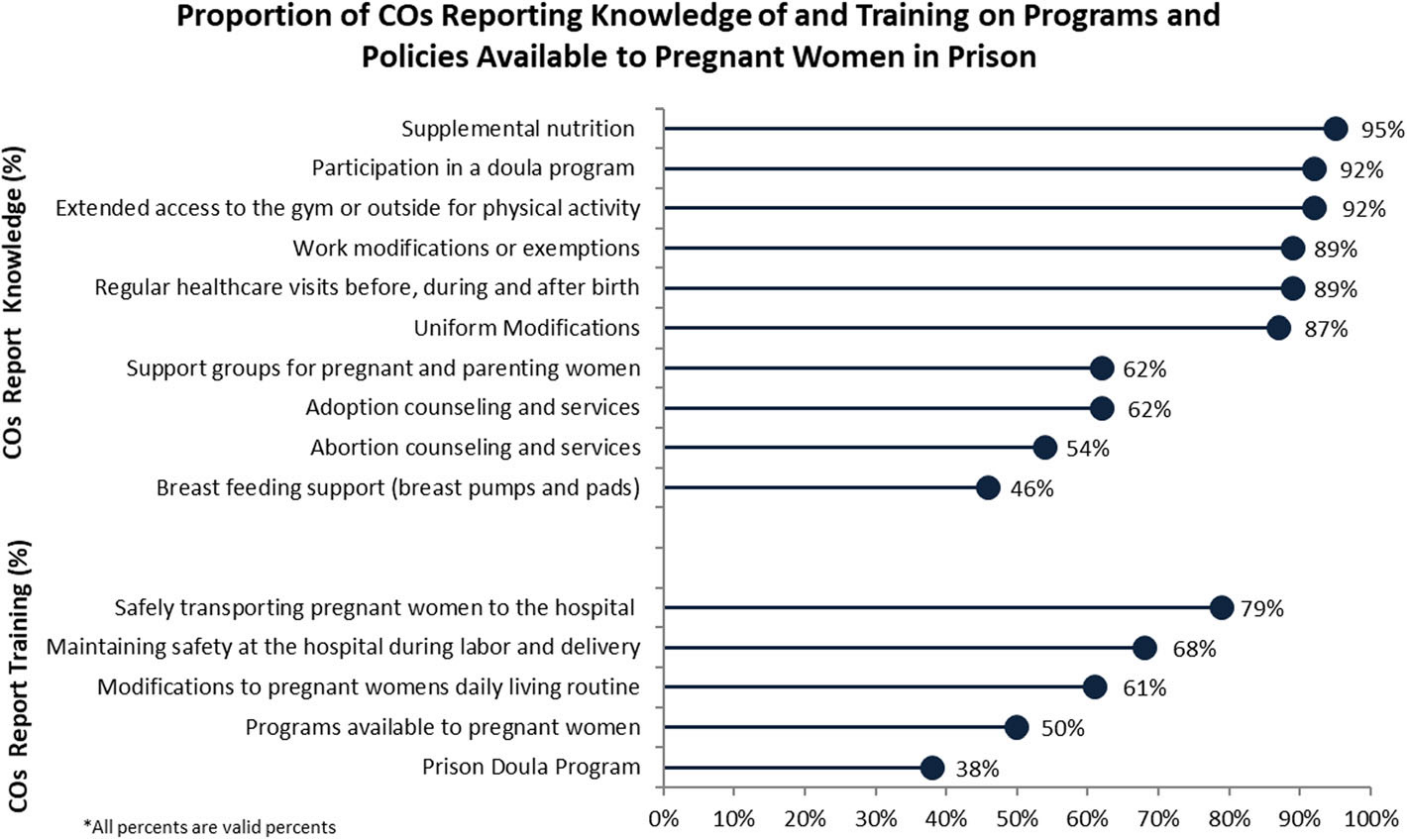
Proportion of CO’s reporting knowledge of and training on MCH programs and policies available to pregnant women in prison

In the survey, COs were asked if they had received training about select topics specific to meeting the unique needs of pregnant women in prison (see Fig. 1). A majority of COs responded that they received training about safely transporting pregnant women to the hospital for medical appointments and delivery (79%) and training on maintaining safety at the hospital during labor and delivery (68%). Fewer COs responded they had received training on the programs specifically available to pregnant women (50%) and fewer than half reported they had received information or training about the prison doula program (38%).

### Survey participants’ perceptions of policies and programs for pregnant women

Most COs had favorable perceptions of the treatment pregnant women received at the prison. A majority (76%) of COs agreed or strongly agreed that the prison’s “policies regarding the treatment of pregnant offenders are comprehensive;” 82% agreed or strongly agreed that the prison’s “policies regarding the treatment of pregnant offenders are fair.” COs generally perceived the health care at the prison to be high quality; 84% of COs agreed or strongly agreed that the prison “provides the same standard of care or better care for pregnant offenders as the care non-incarcerated women would receive.”

The survey results indicated that COs held mixed views about the type of treatment pregnant women should receive compared to non-pregnant women. About half (45%) of COs agreed or strongly agreed that “pregnant women should not be treated any differently than other women in prison;” in contrast, about one-third (34%) disagreed or strongly disagreed with this statement. Similarly, COs were divided on their responses to the statement “I believe pregnant offenders deserve special accommodations in prison,” with 42% expressing agreement and 40% expressing disagreement. Despite these mixed views about the general treatment of pregnant women in prison, COs expressed support and positive perceptions of specific policies and programs that accommodate the needs of pregnant women. For example, the majority of COs (76%) disagreed or strongly disagreed that pregnant women should be restrained during labor and delivery. COs also expressed generally positive perceptions of the prison doula program’s impact on pregnant women, infants, and COs themselves (see Fig. 2)*.*

**Fig. 2:**
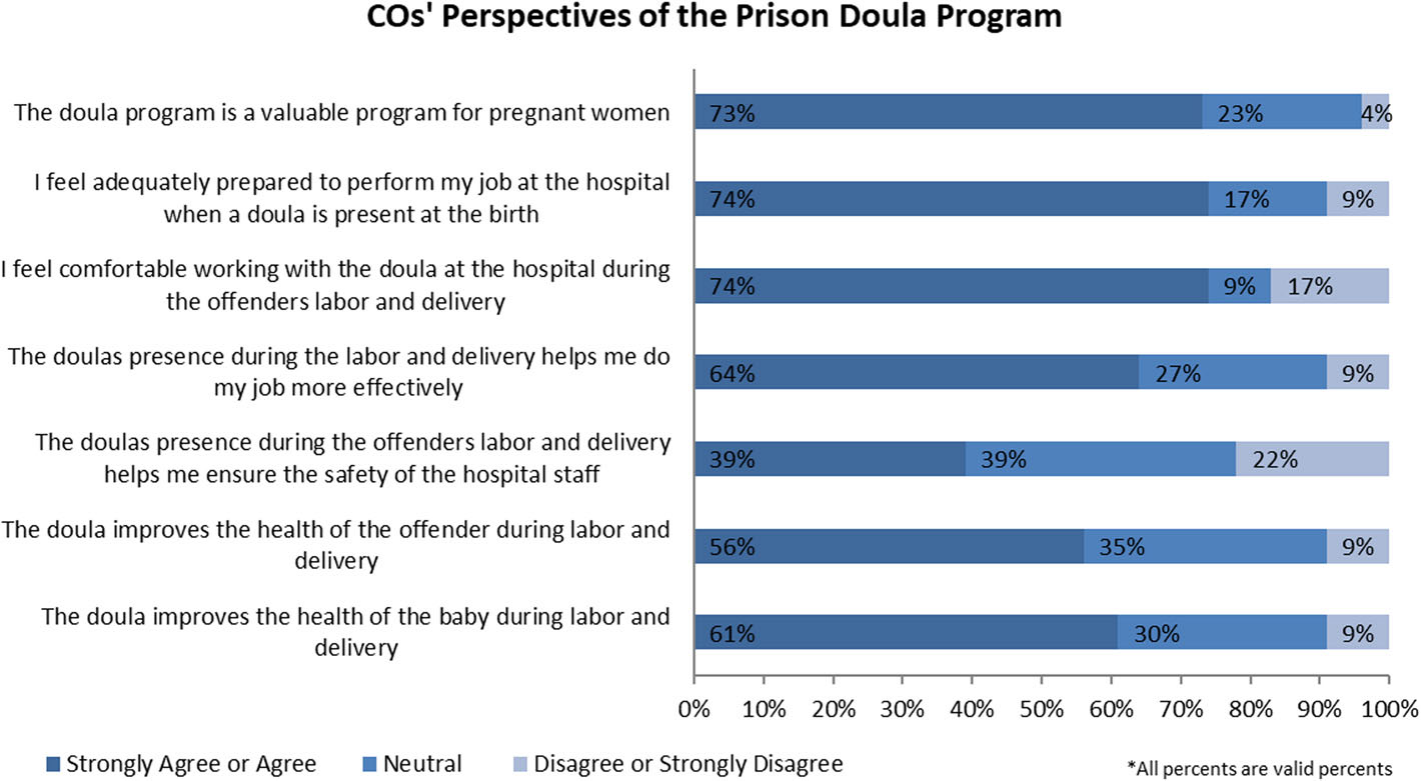
*COs’ perspectives of the prison doula program*

### Qualitative themes from interviews

From the interviews, five major themes regarding COs’ perceptions of MCH policies and programs were identified: 1) COs recognized that pregnancy poses a unique challenge to maintaining professional boundaries in prison; 2) COs perceived the prison doula program as benefitting pregnant women, infants, and their own work as COs; 3) Lack of training about the prison doula program made COs’ jobs more difficult; 4) COs had positive perceptions of the policy prohibiting the use of restraints on pregnant women in addition to concerns about policy implementation; 5) COs’ expressed varied perceptions of health services available to pregnant women.

#### COs recognized that pregnancy poses a unique challenge to maintaining professional boundaries in prison

COs recognized that pregnant women in prison have different needs (e.g. healthcare, physical, and emotional) from the general prison population. A commonly expressed view was that the isolation from social support, lack of physical comforts, and separation from their infants after giving birth were all unique and especially difficult conditions for women in prison. For example, one officer said, “I think it’s tough…they don’t really have a lot of support systems like you would on the outside.”

COs expressed empathy for pregnant women in prison, and most described a “natural” desire to offer them support. Exemplifying this feeling was one CO who said “I believe it is natural to feel empathy for someone who just gave birth to be separated from their child.” These feelings occurred throughout the pregnancy—were heightened during labor and delivery—and often blurred personal and professional boundaries. One officer said, “I mean it’s awkward because as one human to another … there’s a natural want to comfort somebody.” Another female officer remarked, “you really have to try to start to separate your emotions...which is hard for us, especially as women, and if we’re mothers and wives...well that could be your child or that could be you.”

All of the COs who participated in the interviews stressed the need to maintain professional boundaries with women in prison—a boundary that also applied to pregnant women. COs explained that providing emotional and physical support to pregnant women crossed professional boundaries and led to conflict with their primary job task of maintaining safety and security. This tension put them in an “awkward” and “unfair” position, especially in the setting of the delivery room, as they worked to maintain professional distance. One officer said, “There’s a natural barrier for me, where I can’t empathize with the offenders past a certain point, past a point that for me feels like a breach of professionalism.” Another CO stated, “Anybody with any compassion wants to do something for her ‘Can I get you anything? Can I do anything?’, but in our job capacity we, I, shouldn’t be doing anything.”

COs expressed that, in part to maintain professional boundaries, they did not treat pregnant women differently in day-to-day interactions other than straightforward accommodations to women’s physical needs. Interviewees commonly expressed statements such as, “I don’t generally treat [pregnant women] any differently than I would any other offender,” and “I don’t say it really differs, I mean, of course you have to accommodate their physical needs.”

#### COs perceived the prison doula program as benefitting pregnant women, infants, and their own work

The challenges of maintaining professional boundaries with pregnant women were mitigated by MCH programs and policies, and COs expressed appreciation for these programs and policies at the prison, particularly the prison doula program. COs perceived that doulas provided necessary physical, emotional, and moral support to pregnant women who experienced labor and delivery without family or friends. COs with longer tenures reflected on the experience of attending births at the hospital prior to implementation of the prison doula program in 2010; they described feeling pulled between providing emotional support and their job duties of maintaining security at the hospital. Two COs expressed the common sentiment that doulas filled this gap in services: “I think it’s less stressful for staff when [the doulas are] up there because it gives the offender somebody to have for support so that offender isn’t trying to get that support from staff,” and “I cannot be emotionally involved with the offender, so it’s good that [the doulas] are doing it.”

Doulas mitigated professional boundary conflicts by supporting pregnant women in ways that were outside of COs’ job responsibilities so that COs were better able to focus on their security duties. One CO explained, “it takes focus off of how comfortable is the offender, what can I do...it puts my focus back on my security.” COs perceived the doulas made pregnant women feel more supported and comfortable, which led to less volatile situations. As one CO explained, “We have a happier, better cared for offender, that makes our job easier, across the board for the most part.”

COs generally had positive perceptions of the prison doula program and identified benefits to the women, their infants, and the COs themselves. Multiple COs endorsed the program with statements such as “I would say it’s one of the state programs that’s worth holding onto when it comes to pregnant offenders.” COs perceived that the prison doula program benefited both pregnant women and infants by giving them a healthy start to life. One CO explained they believed infants benefited by explaining “If mom’s healthy and happy, then baby’s probably healthy and happy.” Multiple COs also described the prison doula program as a proactive program that not only benefited women and infants, but also benefited the prison in the long run. One CO said, “I think it prevents a lot of problems for the facility because it provides on the front end, it’s proactive versus reactive for the offenders … in the long run it’s a good thing.”

#### Lack of training about the prison doula program made COs’ jobs more difficult

While COs had positive perceptions of the prison doula program, they also expressed that a lack of training on the program added to their job demands and stress. COs stated that they had no knowledge of, or input into, the prison doula program when it began at the prison in 2010. Most COs expressed surprise or confusion regarding their first interactions with doulas, such as “I say initially no [I did not receive training], I kinda showed up and ‘who is that’? Is she supposed to be here? You know, she’s not in scrubs, what’s going on?’”

In the absence of formal training, most COs explained that they learned about the prison doula program “on the job.” One CO explained that they learned about the program through another more experienced CO: “luckily the officer that went up with me, she knew, and then she explained it to me.” Other COs reported being left on their own to understand both the role of the doula and their role as a CO in relation to the doula, “I’ve just learned through experience and trying to use my best judgement.”

This lack of training added awkwardness, uncertainty, and stress at the hospital. COs were concerned about what the doulas were and were not allowed to do; as one CO explained:

*With new staff, if they're not exactly sure what [the doula’s] role is, then you know their mind is more security based, you know from the get-go, so they’re, you know, 'what’s she doing?' now they're trying to watch her too.*

During the interviews, COs commonly requested formal training and information on the prison doula program, specifically the role and activities of the doula at the hospital. COs reported uncertainty on the specific items doulas were allowed to bring in (e.g., essential oils), the protocol for doulas’ taking and sharing pictures, doulas’ use of cell phones with the pregnant women, and physical touch with the pregnant women. The statement below from one CO echoed the sentiment of most of the COs interviewed:

*I think maybe it would be awesome if staff, and maybe I'm out of turn here and maybe it has been done, but if staff could have a class on exactly what the doulas do, and what they're allowed to do, so that every time a new staff goes up with an officer, or with an offender, and the doula comes in they're not wondering, and they're not like 'you can't do that’.*

#### COs had positive perceptions of the policy prohibiting the use of restraints on pregnant women, in addition to concerns about policy implementation

Generally, COs expressed that policies that prohibited the use of restraints on pregnant women met women’s unique physical needs and did not interfere with COs’ role in maintaining security. One CO described how the policy reduced concerns about restraints causing medical issues for pregnant women:

*I think it's helped a lot. Because I think it's alleviated a lot of stress for officers...just the peace of mind I guess, you don't want anybody falling or getting hurt or there being an emergency situation...but you just kind of have that peace of mind that they're not restrained so it's not on me.*

Another CO supported the policy because they felt discomfort restraining women in the hospital who were not deemed a security threat. They explained:

*It makes it easier because it, nobody, I mean, you think I'm comfortable you know cuffing a pregnant woman?....We're being viewed as these brutal people you know and the woman just [gave] birth and so no, it's good for us. I think the changes are great.*

However, uncertainty around consistent and universal enforcement of the policy regarding not restraining pregnant women created confusion and stress for some officers. Some COs, especially those who did not typically interact with pregnant women in their job role, suggested that changes to the policy over the years were not communicated in a systematic way to all COs. One CO expressed frustration with the communication of policy changes: “You have to stay current on how are we doing it now?” and another said the policy itself was “too confusing.” Additionally, COs reported there were not systems in place at the prison to communicate which women were pregnant, which could lead to unintentionally restraining a pregnant woman, especially early in her pregnancy. One CO expressed fear of discipline in these situations, “You handcuff a pregnant offender, you’re going to be investigated” and explained that the fear of discipline caused stress. While most COs expressed strong support for the practice of not restraining pregnant women, a lack of clear communication and training about policy changes and uncertainty about exceptions and special circumstances led to negative perceptions and stress for some COs.

#### COs’ expressed varied perceptions of the health services available to pregnant women

Most of the COs agreed that the health care pregnant women received at the prison was high quality and comparable or better than the care that women would have received in the community. Some COs expressed beliefs that this population of vulnerable, medically underserved women would likely not have access to adequate care in the community. As one CO explained “I think a lot of ‘em receive more than they would on the outside.”

Several COs discussed their perception that the instability of many of the women’s lives prior to coming to prison, especially substance use, meant that the women often received very limited prenatal care in the community. This perception was reflected in statements such as “They absolutely get phenomenal health care, they absolutely have better health care here”, and

*Probably without a doubt this is probably—for I don't know what percentage I can give you—the only care that they get if they go to a doctor at all, and probably the best care that a lot of them would ever receive.*

On the extreme end of COs’ positive health services perceptions, three COs expressed a belief that some women intentionally came to prison to access prenatal care. COs phrased these comments in juxtaposition to their perception that vulnerable women may slip through cracks in the community’s prenatal care system. One CO stated:

*I think, honestly, that they like being here, that they prefer being in [the prison] than being at home cause they get all the health care that is required for pregnant women...some of them told me that 'I just came here cause I got pregnant and I figured that was the only way that I could get medical attention that I needed was to come here.’*

The perception of high-quality health care in prison was not uniformly shared. Some COs stated that the health care was of lower quality because of the extra coordination that off-site medical appointments involved. COs expressed this belief with statements such as “I think high quality is too strong of a phrase, I’m glad they receive care but I don’t think they get as much care as they would if they were free to come and go” and “I don’t think we have enough medical staff here.” Other COs described the care as “comparable” to what women received on the outside. Overall, COs held varied and complex perceptions of the health care pregnant women received in prison.

## Discussion

To our knowledge, this mixed-methods study is the first to examine COs’ understanding and perceptions of MCH policies and programs available to pregnant women in prison, including a prison doula program. We identified five themes from the qualitative interviews that, taken together with the survey results, create a robust narrative of COs’ knowledge and perspectives of MCH services in prison. Results indicated that COs have varied levels of knowledge on the MCH policies and programs available to pregnant women. COs reported high awareness of supplemental nutrition, the prison doula program, extended recreation, work modifications, prenatal health care services and uniform modifications being available to pregnant women, but they were less aware of the adoption and abortion services and breastfeeding support. COs’ perspectives on MCH policies and programs were generally positive, especially regarding the prison doula program and the policy of not restraining pregnant women. COs who completed the survey had differing attitudes on whether pregnant women deserved special accommodations and treatment compared to non-pregnant women in prison. The interviews lent understanding to these divided responses; COs recognized that pregnant women had unique needs, but they generally tried to not treat pregnant women differently because it potentially conflicted with their duty to maintain security.

COs in this study described feeling role conflict—tension between the custodial responsibilities of the job and providing human services and rehabilitation services to people in prison—in their interactions with pregnant women (Aiello 2013; Armstrong and Griffin 2004; Finn 1998; Halsey and Deegan 2017; Misis et al. 2013). They reported that this tension was especially heightened during labor and delivery. Our study is consistent with previous research on COs (Aiello 2013; Aiello 2016; Armstrong and Griffin 2004; Bartels and Gaffney 2011; Halsey and Deegan 2017; Schroeder and Bell 2005a, b). Bartels and Gaffney (2011), for example, found that COs experience role conflict and heightened stress when they are asked to incorporate human services work while also maintaining strict security protocols. In our study, COs with longer tenures recalled that many women lacked emotional and physical support during labor and delivery prior to the implementation of the prison doula program. COs perceived that doulas provided the physical, emotional, and psychological support that women in labor needed while allowing COs to remain focused on their primary job responsibility of maintaining security, which reduced role conflict. Our results are similar to Schroeder and Bell’s (2005a, b) findings that COs strongly approved of a jail doula program in Washington. Our findings add to the small but growing body of evidence that implementation of doula programs in carceral facilities have CO support and may reduce job demands and stress for COs. Ultimately, the findings from this study suggest that MCH policies and programs for pregnant women that also benefit COs by reducing role conflict may lead to higher CO approval and willingness to implement (Shaw et al. 2015).

Lack of CO input during the development and implementation of the prison doula program, and little training since the program began, may have contributed to confusion about safety protocols regarding the doulas in the hospital room (e.g., items allowed in the delivery room). In the survey, fewer than half of COs reported they had ever received training on the prison doula program. As illustrated in the quotes above, COs’ first interactions with doulas at the hospital were often a surprise and many COs described this adding stress to the already chaotic hospital environment. Previous research has shown that increasing officers’ job demands has negative impacts on both COs’ individual health and organizational stability and safety of the prison (Armstrong and Griffin 2004; Finn 1998; Lambert et al. 2018). Based on these findings, we recommend that carceral facilities provide training and opportunities for CO feedback about MCH programming, as well as clear written policies regarding these programs.

Restraining or “shackling” pregnant women is banned in all federal facilities (H.R.5682 - FIRST STEP Act n.d.). As of 2018, however, only 22 states and Washington, D.C. had state laws that prohibit the use of restraints on pregnant women in state and county facilities (Ferszt et al. 2018; King 2018). The current study demonstrates CO support for not using restraints on pregnant women before, during, and in the days after birth. Less than one-quarter of COs who were surveyed agreed that pregnant women should be restrained during labor and delivery. Interviewees indicated that the prison’s policy prohibiting restraining pregnant women did not interfere with their ability to maintain safety and security. COs with longer tenures discussed the positive impact of the state’s anti-shackling policy (passed in 2014) on their job; they described the policy as decreasing their job stress and reducing concerns over potential medical emergencies.

COs held varied and complex perspectives on the quality of health care services pregnant women received at the prison. The majority of COs in the survey agreed that pregnant women at the prison received the same standard of care or better than non-incarcerated women would receive in the community. Interviewees expressed a range of beliefs about the health care women received; some COs felt that the prenatal care in prison was far better than what pregnant women would receive in the community, while other expressed that the inherent restrictions of freedom in the prison setting reduced the quality of health care available. On the extreme end of COs’ positive perceptions of health services, three COs who participated in the interview expressed the sentiment that women in prison preferred being in prison compared to being in the community, because of the prenatal health care available in prison. We do not know how commonly held this perception is among COs at the prison who did not participate in an interview, but past research has documented similar perceptions by COs and medical security staff in other carceral facilities (Sufrin 2017). Yet, past research with women who have given birth while incarcerated suggests that women do not share the same positive perceptions, reporting that the prenatal care they received was of low quality, being restrained was dehumanizing, and separation from their infants after birth was a traumatic experience (Fritz and Whiteacre 2016; Schroeder and Bell 2005a, b; Williams and Schulte-day 2006; Wismont 2000). Additionally, research with prison administrators and national reviews of prison policies suggests that the majority of prisons are not meeting the prenatal and postnatal needs of pregnant women (Kelsey et al. 2017; Ferszt and Clarke 2012; Shlafer et al. 2019).

Similar to other researchers, our findings illustrate the complexities of health care available to women from marginalized communities, including the role of prisons in providing needed care to pregnant women (Baldwin et al. 2018; Cross 2019; Shaw et al. 2015; Sufrin 2017; Sufrin et al. 2019). Sufrin’s research (2017) highlights how prisons can become a protective place for some pregnant women; women who are homeless, living with mental illness, or using substances may struggle to access adequate prenatal care in the community. Prison may present a unique opportunity to minimize risk factors and provide MCH services inaccessible to women outside of the carceral system, such as prenatal health care, basic nutrition and shelter, a reduction in high risk behaviors such as substance use, and separation from domestic abuse situations (Baldwin et al. 2018; Shaw et al. 2015; Sufrin 2017). Some research has shown that prisons with enhanced MCH policies and programs may have a protective effect on certain clinical pregnancy outcomes, such as infant birth weight (Bard et al. 2016). COs in this study often cited these factors when describing their perceptions of the care available at the prison to be of higher quality compared to the availability of services in the community.

However, focusing only on the protective aspects of prison ignores the broader social and structural health determinants that affect people in prison and their children before, during, and after their incarceration, including violence in prisons and the trauma that occurs when mothers and infants are separated after birth (Sufrin et al. 2019; Shaw et al. 2015). The reality is that many prisons in America have become de facto social service providers due to inadequate mental health, substance use, and social services available to marginalized pregnant women in the community (Fearn and Parker 2004; Sufrin 2017). Viewing prisons as “protective” also ignores the history of reproductive control and coercion experienced by racial/ethnic minority women: from slavery’s manipulation of Black women’s fertility for economic benefit, to forced sterilization campaigns in the twentieth century, to the modern-day disproportionate representation of minority women in the US criminal justice system - a system where reproduction is highly regulated (Roberts 1997). In the current study, we explored these issues of reproductive justice using MCH and public health frameworks. However, the officers’ language (e.g., use of “offender”) and their perceptions of the prison environment and their roles, could be considered with sociological theories, including Foucault’s concepts of surveillance (Foucault 1983) and Goffman’s assessment of the master status (Hunt 2007). This is a valuable area for future inquiry and using a sociological framework would contribute to a richer understanding of these complex issues.

### Limitations

The current study had several limitations. The small sample size and low (28%) response rate to the survey, as well as the small convenience sample of COs who participated in an interview limits the generalizability of this study and may not be representative of all COs at the prison. Further, our study only included COs at one women’s state prison, limiting generalizability. Based on their role in prison, the participants varied in the amount of direct contact they had with pregnant women and it is unknown how COs’ knowledge and perceptions may differ based on these roles. Compared to COs who participated in the survey, COs who participated in the interviews were more likely women and older. As such, COs qualitative responses may not reflect all the COs at the prison. Furthermore, the small number of interviews may not have allowed us to reach saturation of themes. Additionally, knowing that the results from the study would be disseminated could have accounted for socially desirable responses.

### Recommendations and conclusion

As the largest occupational group in prisons, COs’ unique perspectives should be incorporated when creating and evaluating MCH programming. Safety and security is a priority for prison administrators and COs. Therefore, in order for MCH programming to be considered, implemented, and successfully sustained in more women’s carceral facilities, further research should build upon this study to examine how reducing CO work stress, helping COs maintain professional boundaries, and allowing COs to remain focused on their primary task of safety and security, MCH services may improve conditions for both COs and women in prisons. This future work should be done in concert with research examining incarcerated pregnant women’s perspectives of the services they need, as well as community-based alternatives to incarceration. To reduce the challenges and stress that some COs described experiencing in their work with pregnant women, implementing future MCH programs and policies with robust training and opportunities for CO input will help ensure that programming optimally supports all key stakeholders. Community-based programs that enter carceral spaces to deliver MCH programming, such as prison doula programs or parenting support groups, may consider having a CO “champion” within the facility to provide CO perspective and have a point person to help lead trainings. Clear, written policies for MCH programs and policies is an integral first step to ensuring COs can perform their jobs effectively. Given the high rate of incarcerated women in the US, MCH policies and programs in prisons and jails are needed to support the unique health care needs of pregnant women. Ultimately, incorporating COs’ perspectives into the development and implementation of MCH programs and policies in prisons may both improve facility safety and promote maternal and child health.

## Data Availability

While all efforts were made to remove any potentially identifiable information from the collected data (i.e., personal identifiers) it is possible, given the small sample size, that indirectly identifiable information could be gleaned from the collected data sets. As such, the data sets will not be shared.

## Abbreviations

CO: Corrections Officer
DOC: Department of Corrections
MCH: Maternal and Child Health
MnPDP: Minnesota Prison Doula Project
